# Proteomics-Based Investigation of Different Live Prey Administered to Freshwater Dark Sleeper (*Odontobutis potamophila*): Examining the Effects on Glycolipids and Energy Metabolism

**DOI:** 10.3390/metabo14020085

**Published:** 2024-01-24

**Authors:** Zihan Zhou, Qichen Jiang, You Zheng, Chen Hao, Shuyan Ding, Mengya Guo, Yunlong Zhao, Guoxing Liu, Shuyan Miao

**Affiliations:** 1 College of Animal Science and Technology, Yangzhou University, Yangzhou 225009, China; 2 Freshwater Fisheries Research Institute of Jiangsu Province, Nanjing 210017, China; 3 Low-Temperature Germplasm Bank of Important Economic Fish (Freshwater Fisheries Research Institute of Jiangsu Province) of Jiangsu Provincial Science and Technology Resources (Agricultural Germplasm Resources) Coordination Service Platform, Nanjing 210017, China; 4 School of Life Science, East China Normal University, Shanghai 200241, China

**Keywords:** proteomics, glycolipid metabolism, *FAS*, *Odontobutis potamophila*, *GK*

## Abstract

Live prey is characterized by balanced rich nutrients and high palatability and is widely used for the seedling cultivation of freshwater dark sleeper (*Odontobutis potamophila*) larvae. In this study, we evaluated the effects of four groups of paired feeding regimens (group C (*Daphnia magna*), group L (*Limnodrilus hoffmeisteri*), group H (*Hypophthalmichthys molitrix* fry), and group M (mixed groups C, L, and H)) on glycolipid and energy metabolism in *O. potamophila* larvae. We observed that fatty acid synthase (*FAS*) and sterol-regulatory-element-binding protein-1 (*SREBP-1*) mRNA levels were significantly lower in group H when compared to mRNA levels in the other three groups (*p* < 0.05) and that carnitine palmitoyltransferase 1α (*CPT1-α*) mRNA levels were significantly lower in group L when compared to group M (*p* < 0.05). Relative glucokinase (*GK*) expression levels were significantly lower in group M when compared to the other three groups (*p* < 0.05). Using proteomics, we analyzed and compared groups H and L and identified 457 differentially expressed proteins (DEPs), of which 151 were significantly up-regulated and 306 were significantly down-regulated. In the comparison of group M with groups C, L, and H, we found significant enrichment in glycolytic processes, the endoplasmic reticulum lumen, NAD binding, intermediate filaments, and nutrient reservoir activity. Our results provide a theoretical guidance for bait selection during larvae cultivation stages in carnivorous fish.

## 1. Introduction

As a major component of bait food, live prey, when compared to compound feed, contains a variety of species, has high resistance, and ensures rapid reproduction, easy selection, and breeding qualities [1,2]. Live prey feed does not contaminate water quality and the environment, has balanced and rich nutrition, and has high palatability, all of which are largely absent in compound feed [3,4]. Specifically, for cultured fish fry cultivation stages, live prey selection is particularly important [3,5,6].

To meet the needs of cultured animals, live prey is available in many sizes and nutrient forms [7,8,9]. It was previously reported that when fish larvae ingested compound feed, they died when their intestines were full of food [5,10], which indicated that the larvae could not digest compound feed well and they had insufficient intestinal digestive enzymes [11]. Therefore, an urgent need exists to generate exogenous enzymes from live prey. Due to small orifice sizes and slow movements, live prey is mostly used to satisfy nursery-stage requirements of cultured fish [1,12]. As live prey is rich in intestinal neuropeptides and nutrient growth factors, it enhances digestive capacity; thus, live prey is superior to compound feed [13,14].

Live prey is currently used in domestic and international production and mainly includes the following categories: plant-based baits, such as *Chlorella*, *Microchlorophyceae*, *Spirulina,* and photosynthetic bacteria [15]; animal-based baits, such as *Rotifera*, *Halobacterium*, *Cladocera*, and *Tubifex* [16]; and live prey, such as fish fry and mysid shrimp. Currently, selecting optimal and palatable live prey is a key factor in the cultivation stages of aquaculture species that only eat live prey [2], which may significantly affect the growth status and market efficiency of aquaculture animals.

The freshwater dark sleeper (*Odontobutis potamophila*) is a well-known economic freshwater fish with a unique diet and lifelong feeding on live prey [17]. Currently, the most commonly used live prey in production are Cladocera, Tubifex, and silver carp fry. Therefore, we selected four bait combinations that are highly economically and profitable and are favored by *O. potamophila* larvae on the market, namely Cladocera (*Daphnia magna*), Tubifex (*Limnodrilus hoffmeisteri*), and silver carp (*Hypophthalmichthys molitrix*) fry and a combination of these baits in a 1:1:1 mix. These characteristics generate high-nutritional-value and good-quality *O. potamophila* meat, which suggests good aquaculture prospects. Cladocera (*D. magna*) is recommended for *O. potamophila* larvae feeding as it has a good nutrient content and specifications for *O. potamophila* [18,19]. *L. hoffmeisteri* has a high protein content and outstanding locomotor ability [20,21], which provide strong feeding stimuli for *O. potamophila* larvae. *H. molitrix* has a high unsaturated fatty acid content [22], which is suitable for intensive *O. potamophila* larvae cultivation. Considering the application of the final solution to production practice, we also mixed the three baits to explore their combined effects on *O. potamophila* larvae. We additionally selected the most representative genes for glycolipid metabolism for the assay, such as sterol-regulatory-element-binding protein-1 (*SREBP-1*), *carnitine palmitoyltransferase 1α* (*CPT1-α*), *phosphoenolpyruvate carboxykinase* (*PEPCK*), and *glucokinase* (*GK)*.

However, few studies have investigated *O. potamophila*; therefore, an urgent need exists to identify the selection and breeding of good bait for *O. potamophila* fry breeding stages. In this study, we investigated the effects of different live prey on in vivo glycolipid metabolism in *O. potamophila* larvae, with a view to providing theoretical guidance for live prey selection for larvae-stage culture.

## 2. Materials and Methods

### 2.1. Experimental Conditions

Studies were conducted at the Yangzhong site of the Freshwater Aquatic Research Institute of Jiangsu Province, China. We have bred *O. potamophila* parents at our base in recent years, and we used our base facilities to transfer the eggs of the parents for indoor culture till the *O. potamophila* larvae grew to the appropriate length to start the experiment. The *O. potamophila* larvae were temporarily reared in nets for 1 week before the start of the formal trial, which consisted of four different treatment groups with five replicates/treatment. Two thousand *O. potamophila* larvae (initial mean weight = 0.11 ± 0.01 g) were stocked in 30 L nets, each containing 100 larvae. Each net was made of a 100-mesh sieve, and one group of nets was placed in a large 3000 L water tank with drainage holes. Treatments provided the corresponding diets to larvae in the five replicate nets for the study duration (56 days). Water was changed daily during the test period to approximately one-third of the total net box volume, keeping the water free of debris and residual bait. Water quality was maintained at stable levels, where water parameters were temperature ranging from 22.1 °C to 26.9 °C, pH 7.5–8.4, and dissolved oxygen content > 6 mg/L. We fed the *O. potamophila* larvae until there was no bait left in the experimental nets, and diets were adjusted weekly based on growth, feeding response observations, and mortality rates.

### 2.2. Experimental Diets

All larvae diets consisted of live prey. In group C, we used *D. magna* fortified with an amino acid concentrate; in group L, *L. hoffmeisteri* was the main ingredient; in group H, *H. molitrix* was used as bait; and in group M, on a quality basis, we used a 1:1:1 mixture of the aforementioned diets. We weighed the 1:1:1 bait before feeding to make sure that we were feeding equal portions each time. Before conducting formal experiments, *D. magna* and *H. molitrix* fry, which had been cultured and fortified with a supplemental amino acid concentrate (mainly lysine, methionine, arginine, etc.) in two hatcheries, were bred by the Jiangsu Institute of Freshwater Aquatic Research (refer to our factory farming guidelines). Fresh bait was provided daily to feed *O. potamophila* larvae in sufficient quantities at 8:00 and 18:00, and diets were adjusted weekly based on growth, feeding response observations, and mortality rates.

### 2.3. Sample Collection

A liquid nitrogen tank was prepared before study end (56 d). Larvae were randomly selected from each group, fished out, and washed in distilled water, followed by rapid sealing in frozen tubes and immersing in liquid nitrogen for metrics analyses. The growth performance of *O. potamophila* larvae was measured with the growth parameter index, the weight gain rate (WGR), and the specific growth rate (SGR). These indicators were calculated according to the formulae:

WGR (%) = 100 × (W_f_ − W_i_)/W_i_

SGR (%/day) = 100 × (ln W_f_ − ln W_i_)/T

Survival rate (%) = 100 × N_i_/N_f_

where W_f_ is the final weight of *O. potamophila* larvae, W_i_ is the initial weight of *O. potamophila* larvae, T is the feeding day when the samples were collected, N_i_ is the initial number of *O. potamophila* larvae, and N_f_ is the number of samples of *O. potamophila* larvae.

### 2.4. RNA Extraction and Sequencing

RNA was extracted from larvae for real-time (RT) fluorescence quantitative polymerase chain reaction (PCR) analysis. Total RNA was reverse-transcribed into cDNA using a Prime Script RT reagent kit (TaKaRa, Shiga, Japan) and stored at −80 °C. RT–quantitative PCR (RT-qPCR) was then performed using CFX96 RT-PCR equipment (BioRad, Hercules, CA, USA) and the Trans Start Top Green qPCR Super Mix (Transgenics, Beijing, China). Ten target genes were selected for qRT-PCR analysis based on the results of transcriptome sequencing, which we have only just sequenced. Sequence reliability was verified. Primers for all 10 target genes were designed using Primer 5.0 (PREMIER Biosoft, Palo Alto, CA, USA), as shown in Table 1. Each primer pair was verified with gel electrophoresis before being used for qPCR. Specific metabolic-related gene primers and sequences are shown (Table 1). The qPCR assays were conducted in triplicate, using the β-actin gene as an internal control to standardize the expression levels of the target genes and group M as the control group. The relative quantification of qPCR data was determined using the 2^−ΔΔCt^ method.

### 2.5. Protein Collection and Isolation

Samples were lysed in 300 μL of RIPA buffer (Cell Signaling Technology, Danvers, MA, USA) plus the protease inhibitor phenylmethyl sulfonyl fluoride (Beyotime, Shanghai, China). Samples were then sonicated on ice for 3 min, and cell debris was separated using centrifugation (12,000 rpm, 10 min, 4 °C). Protein concentrations were measured using a bicinchoninic acid assay protein quantitation kit after 10 μg of protein was separated using 12% sodium dodecyl sulfate–polyacrylamide gel electrophoresis. After eStain LG Protein Stainer (Nanjing Kingsley Biotechnology Co., Ltd., Nanjing, China) staining, images were generated using an automatic digital image analysis system (Tanon 1600; Tanon Science & Technology, Co., Ltd., Shanghai, China).

### 2.6. Protein Digestion and TMT Labeling

Proteins were reduced in 5 mM dithiothreitol at 55 °C for 30 min, alkylated in 10 mM iodoacetamide for 15 min at room temperature in the dark, precipitated in pre-cold acetone at −20 °C overnight, and diluted by adding 200 mM TEAB. Next, a first trypsin (MS grade; Corning, Somerville, MA, USA) digestion step was performed overnight at a 1:50 trypsin-to-protein mass ratio, and a second digestion was then conducted at a 1:100 ratio for 4 h. After the digestion steps, peptides were desalted on a Strata X C18 SPE column (Phenomenex, Torrance, CA, USA) and dried under vacuum. A peptide mixture was reconstituted in 100 mM TEAB from the TMT kit (Thermo Fisher Scientific, Waltham, MA, USA) following the manufacturer’s instructions. Samples were then incubated for 1 h at room temperature and dried under vacuum centrifugation, the reactions were terminated with 5% hydroxylamine addition, and the samples were lyophilized and then finally stored at −80 °C [23].

### 2.7. Liquid Chromatography with Tandem Mass Spectrometry (LC-MS/MS)

An Agilent 1100 high-performance liquid chromatography system (Agilent, Palo Alto, USA). was used for reverse-phase liquid chromatography separation on an Agilent Zorbax Extend-C18 column (2.1 × 150 mm, 5 μm) with UV detection at 210 nm. Mobile phases A and B were ACN-H_2_O (2:98, *v*/*v*) and ACN-H_2_O (90:10, *v*/*v*), respectively, and the flow rate was 300 μL/min. Gradient elution conditions were as follows: 0–8 min, 98% A; 8.00–8.01 min, 98–95% A; 8.01–30 min, 95–80% A; 30–43 min, 80–65% A; 43–53 min, 65–55% A; 53–53.01 min, 55–10% A; 53.01–63 min, 10% A; 63–63.01 min, 10–98% A; and 63.01–68 min, 98% A. Samples were collected between 8 and 54 min. Eluate buffer was collected every minute in centrifuge tubes, numbered 1–15, and cycled in this order until gradient end. After collection, the frozen samples were prepared for MS.

Samples were loaded and separated on an Acclaim Pep Map RSLC 75 μm × 50 cm column (RP-C18, Thermo Fisher, Woburn, MA, USA) on an EASY-nLC 1200 system (Thermo Fisher, Woburn, MA, USA). Gradient elution conditions were as follows: 0–50 min, 2–28% B; 50–60 min, 28–42% B; 60–65 min, 42–90% B; and 65–75 min, 90% B. Mobile phases A and B phases were H_2_O-FA (99.9; 0.1, *v*/*v*) and ACN-H_2_O-FA (80; 19.9; 0.1, *v*/*v*/*v*), respectively. Mass resolution was 60,000, and the automatic gain control value was 3 × 10^6^. The system was set to scan full mass ranges at 350–1500 *m*/*z*, and the 20 highest peaks were identified using MS/MS. All MS/MS spectra were collected using high-energy collisional fragmentation, with collision energy set to MS/MS resolution, automatic gain control, maximum ion accumulation time, and dynamic exclusion time, which were 45,000, 2 × 10^5^, 80 ms, and 30 s, respectively.

### 2.8. Protein Quantification and Bioinformatics Analysis

Data were analyzed using Proteome Discoverer 2.4.1.15 software (Thermo Fisher, Woburn, MA, USA). The UniProt daphniidae database was used to search for proteins, and the false-positive rate of peptide identification was controlled to below 1%. Specific parameters were trypsinization digestion specificity for the database search, cysteine alkylation for fixed modifications, and TMT6-plex for protein quantification. Additionally, missed cleavages were set to 2, MS 1 tolerance was set to 20 ppm, and MS 2 tolerance was set to 10 ppm. According to the Score Sequest HT > 0, unique peptides ≥ 1, and criteria for removing blank values, credible proteins were screened from raw data. Each data group was screened for significant proteins to generate fold-change (FC) values and *p*-values of comparison groups. We used FC > 1.2 or FC < 5/6 standards and a *p*-value of < 0.05 to screen for differentially expressed proteins (DEPs). *R* software (*R* Foundation for Statistical Computing, Vienna, Austria) version 4.2.0 was used for statistical analysis of data, and ggplot2 (3.3.0) was used for image visualization. The normalization method comprised an algorithm found in Proteome Discoverer 2.4.1.15 software.

The Benjamini–Hochberg algorithm was used to adjust *p*-values. Biological function analyses were based on DEPs. To analyze DEP functions, the Omics Bean data integrated analysis cloud platform was used to determine Gene Ontology (GO) functional annotations and Kyoto Encyclopedia of Genes and Genomes (KEGG) enrichment. The method of enrichment analysis used the species protein as the background list and screened a differential protein list as the candidate list. Hypergeometric distribution tests were used to calculate *p*-values, which represented significant functional enrichment in DEP lists. The *p*-values were corrected for false discovery rates using Benjamini–Hochberg multiple testing corrections. GO functional annotations included three analysis categories: biological processes (BP), cellular components (CC), and molecular functions (MF).

### 2.9. Statistical Analysis

The data were checked for homogeneity of variances using the Bartlett test and, where necessary, arc-sin-transformed before further statistical analysis. To determine the differences between the control group (group M) and other groups, one-way analysis of variance (ANOVA) and Tukey’s test were used for the relative mRNA levels of the target genes. Differences were reported as statistically significant when *p* < 0.05. All statistical analyses were performed using SPSS 26.0 (IBM, Armonk, NY, USA). Graphs were drawn in GraphPad Prism 8.0 software (GraphPad, San Diego, CA, USA).

## 3. Results

### 3.1. Growth Performance

During the 56-day experiment, there was no significant death observed in *O. potamophila* larvae fed different live prey. The growth of each group is shown in Table 2. Compared to the other three groups (groups C, L, and H), the FBW significantly increased in group M (*p* < 0.05). In terms of the WGR and the SGR, groups L and M had significantly higher values than groups C and H (*p* < 0.05) (Table 2).

### 3.2. Differentially Expressed Genes

**Figure 1:**
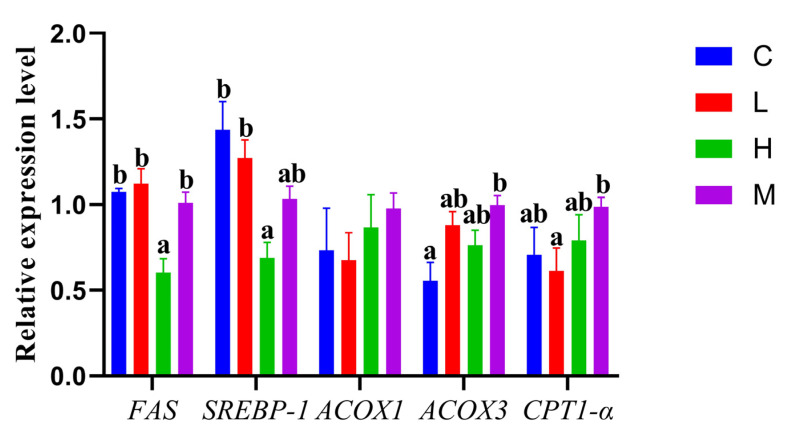
*FAS*, *SREBP-1*, *ACOX1*, *ACOX3*, and *CPT1-α* mRNA levels in *O. potamophila* larvae. Data are represented by the mean ± standard deviation. The same letters over columns indicate non-significant differences (*p* > 0.05). Significant differences from the control group (group M) are indicated by different letters (*p* < 0.05).

**Figure 2:**
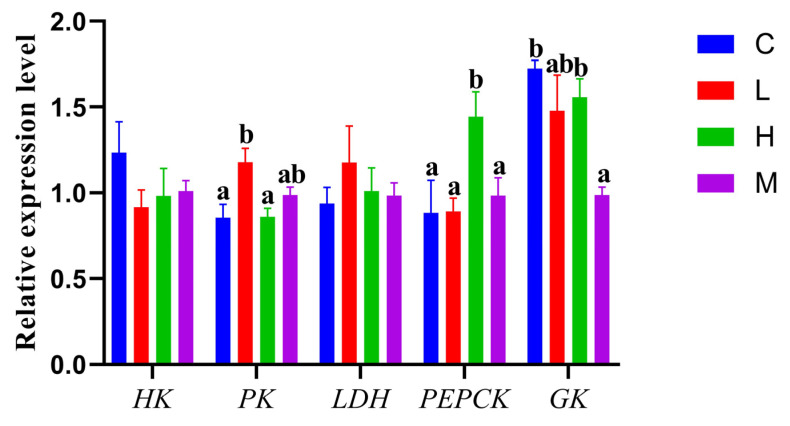
*HK*, *PK*, *LDH*, *PEPCK*, and *GK* mRNA levels in *O. potamophila* larvae. Data are represented by the mean ± standard deviation. The same letters over columns indicate non-significant differences (*p* > 0.05). Significant differences from the control group (group M) are indicated by different letters (*p* < 0.05).

As shown in Figure 1, *FAS* and *SREBP-1* mRNA levels were significantly lower in group H when compared to the other three groups (*p* < 0.05). *ACOX3* mRNA levels were significantly lower in group C when compared to group M (*p* < 0.05). In group L, *CPT1-α* mRNA levels were significantly lower when compared to group M (*p* < 0.05). As indicated (Figure 2), groups C and H had significantly lower relative *PK* mRNA levels when compared to group L (*p* < 0.05). For phosphoenolpyruvate carboxykinase (*PEPCK*) mRNA levels, we observed they were significantly higher in group H than in all other groups (*p* < 0.05), while relative glucokinase (*GK*) mRNA levels were significantly lower in group M when compared to the other three groups (*p* < 0.05), but mRNA levels were significantly higher in groups C and H when compared to group M (*p* < 0.05). 

### 3.3. DEP Identification in O. potamophila Larvae

**Figure 3:**
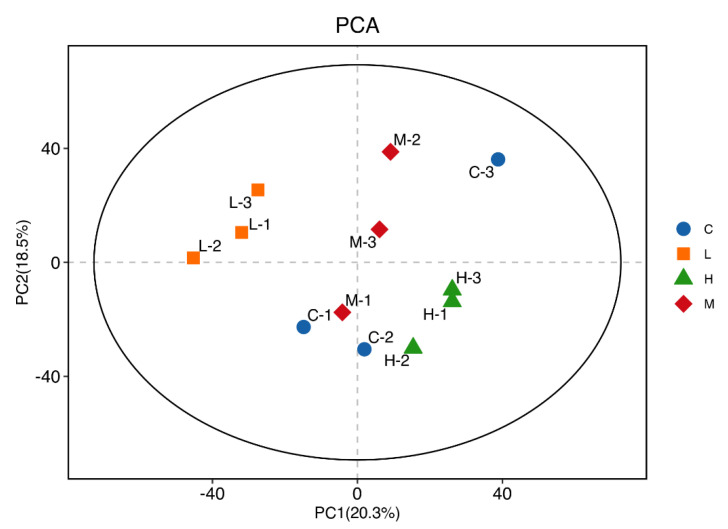
Principal component analysis of differentially expressed proteins in different *O. po-tamophila* larvae groups after live prey administration.

**Figure 4:**
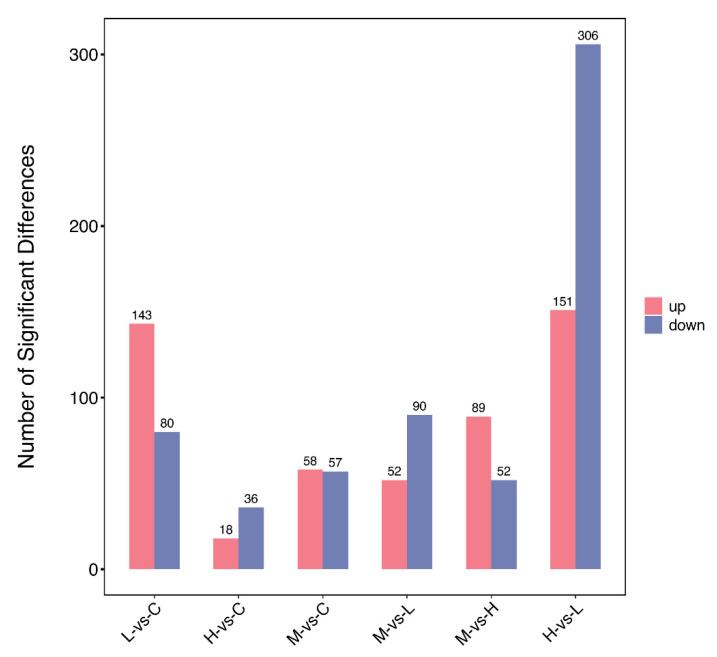
Overall distribution of differentially expressed proteins (DEPs) in *O. potamophila* larvae, with comparison groups on the horizontal axis and DEP numbers on the vertical axis.

**Figure 5:**
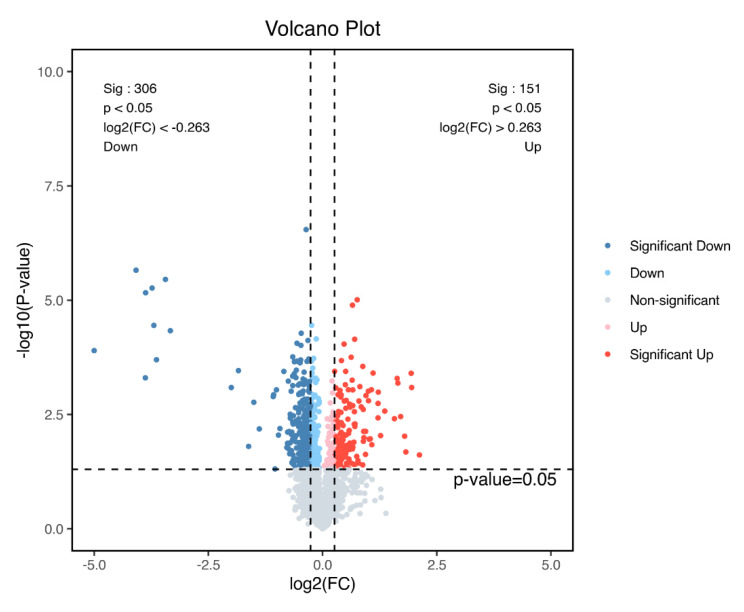
Volcano plot showing differentially expressed proteins (DEPs) in *O. potamophila* larvae: red, up-regulated DEPs; blue, down-regulated DEPs; and light blue, light red, and grey, no apparent DEPs.

Principal component analysis was performed using plausible protein expression (Figure 3), which identified four distinct clusters. Based on these proteins, we compared screened DEPs two by two; as indicated (Figure 4), significant DEP numbers in group H versus group L differed the most. DEPs were screened using log2(FC) > 0.263 or <−0.263 and *p* < 0.05 criteria, and as indicated by the volcano plot (Figure 5), up-regulated proteins numbered 151 and down-regulated proteins numbered 306, with a total of 457 identified DEPs.

### 3.4. Functional Enrichment Analysis of DEPs in O. potamophila Larvae

**Figure 6:**
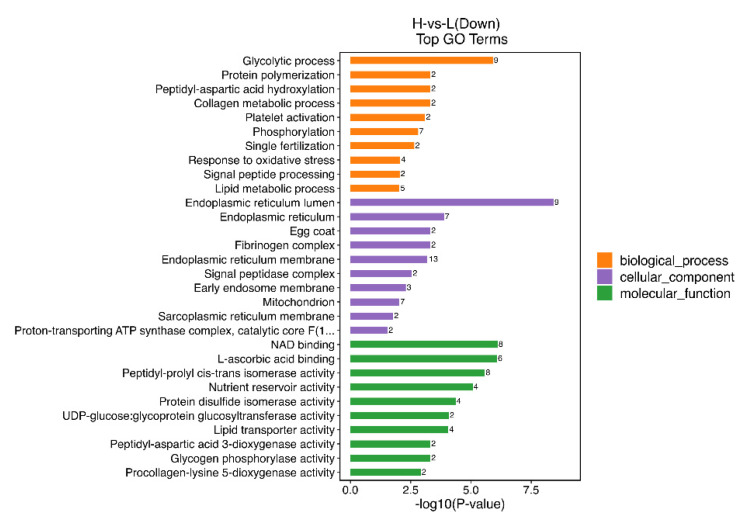
Group H vs. group L (down) showing the top 30 Gene Ontology (GO) terms: yellow, purple, and green colors indicate biological processes, cellular components, and molecular function, respectively. For each entry category, the closer the bar is to the right, the more significant is the difference.

GO functional analysis showed that in group H versus group L (down), DEPs were significantly enriched in the top 30 GO terms (Figure 6), with the most significant effects (*p* < 0.05) observed for glycolytic processes (GO:0006096) in biological processes, the endoplasmic reticulum lumen (GO:0005788) in cellular components, and nicotinamide adenine dinucleotide (NAD) binding (GO:0051287) in molecular function (*p* < 0.05).

**Figure 7:**
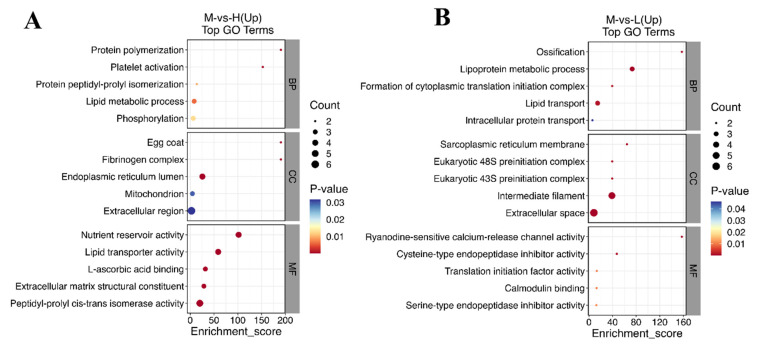
Top Gene Ontology (GO) enrichment terms. The solid circle color and size represent *p*-values and enriched protein numbers in pathways, respectively. (**A**) Group M vs. group H (up); (**B**) group M vs. group L (up).

**Figure 8:**
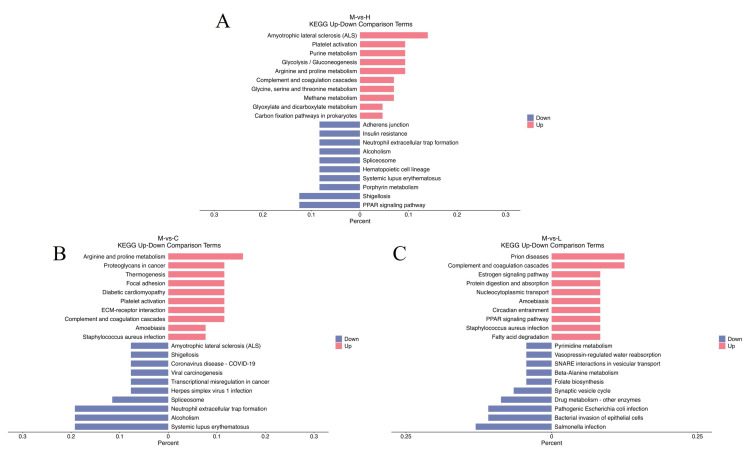
Kyoto Encyclopedia of Genes and Genomes (KEGG) enrichment analysis of up- and down-regulated entries in the 10 pathways with the smallest KEGG up- and down-regulated *p*-values. Horizontal coordinates indicate the ratio of the number of foreground proteins to the number of background proteins of the pathway (list hits/total hits), and vertical coordinates refer to the KEGG pathway. (**A**) Group M vs. group H; (**B**) group M vs. group C; (**C**) group M vs. group L—some groups were significantly enriched in the up- and down-regulation.

In GO bubble plot analysis (Figure 7), we selected two comparison groups with significant differences, namely group M versus group H (up) (Figure 7A) and group M versus group L (up) (Figure 7B). Significant effects were mainly observed for nutrient reservoir activity (GO:0045735) in group M versus group H (up) and intermediate filaments (GO:0005882) in group M versus group L (up). A comparison of up- and down-regulated top entries in KEGG enrichment analyses is shown in Figure 8, where up-regulated DEPs were significantly enriched for amyotrophic lateral sclerosis in group M versus group H (Figure 8A), up-regulated DEPs were significantly enriched for arginine and proline metabolism in group M versus group C (Figure 8B), and up-regulated DEPs in group M versus group L were significantly enriched for complement and coagulation cascades (Figure 8C).

## 4. Discussion

When fish are fed different live prey, many key enzymes and transcription factors are expressed that regulate nutrient metabolism in the body [18,24,25], which is mainly classified into glycometabolism, fat metabolism, and protein metabolism processes. These processes are interrelated and interact with each other, and together, they maintain the energy balance and normal physiological function in the body [26,27].

During the experimental period, the *O. potamophila* larvae all grew well and maintained a survival rate of more than 90%. In our study, we found that the FBW of group M was significantly higher than that of the other three groups. The high growth rate brought about by a comprehensive feeding strategy was consistent with our expectations, and we also observed different regulatory mechanisms in *O. potamophila* larvae in response to the four different feeding strategies. *FAS* is a key rate-limiting enzyme involved in organismal lipid synthesis [28], is mainly involved in fatty acid synthesis [29], and catalyzes acetyl-CoA and malonyl-CoA conversion to long-chain fatty acids in the cytoplasm [30]. *FAS* mRNA levels in larvae fed *H. molitrix* were significantly lower when compared to the other three groups; therefore, we hypothesized this was possibly caused by higher unsaturated fatty acid levels in *H. molitrix,* resulting in insufficient levels of short-chain fatty acids for fatty acid synthesis [31]. The phenomenon of unsaturated fatty acids affecting *FAS* mRNA levels in species is consistent with a previous study in rats [32]. *FAS* mRNA levels are also subjected to *SREBP-1* regulatory effects [33], which are generated from the 125 kDa SREBP-1c precursor protein that is processed by hammerhead proteases and insulin-sensitive proteases, resulting in its translocation from the cytoplasm to the nucleus for activation [34]. In our *FAS* and *SREBP-1* mRNA level analyses in *O. potamophila* larvae, both molecules exhibited roughly the same patterns, with activated *SREBP-1* entering the nucleus, binding to regulatory elements at fixed positions in DNA, and acting directly on the *FAS* promoter region to activate gene transcription related to lipid metabolism [35,36]. *FAS* and *SREBP-1* mRNA levels in group H were significantly lower when compared to the other three groups, further confirming our speculation on *FAS*, consistent with a previous study [37].

In lipolysis processes, *ACOX1* and *ACOX3* are different acyl-coenzyme A oxidases that help maintain balanced fatty acid metabolism [38]. We observed that relative *ACOX1* and *ACOX3* mRNA levels were highest in group M. *ACOX3* is a linoleic acid dehydrogenase and is mainly localized to the endoplasmic reticulum, where it helps maintain fatty acid metabolism and homeostasis [39]. *ACOX3* catalyzes dehydrogenation reactions on linoleic acid and other unsaturated fatty acids [40], thus participating in several lipid metabolism physiological processes [41]. We observed that *O. potamophila* larvae had significantly higher relative *ACOX3* mRNA levels when compared to the other three groups after ingestion of group M.

*CPT1-α* is a key carnitine palmitoyltransferase 1-α subunit that binds long-chain fatty acids to carnitine coenzyme A [42] and allows them to cross the inner mitochondrial membrane into the mitochondria for further metabolism [43]. *CPT1-α* demonstrated similar functions as *ACOX1* and *ACOX3* after *O. potamophila* larvae ingested different live prey; after feeding, up-regulated *CPT1-α*, *ACOX1,* and *ACOX3* mRNA expression levels were identified in group M.

Similarly, in terms of carbohydrate metabolism, a cascade of different metabolic reactions was induced when *O. potamophila* larvae ingested different live prey. Lipids and sugars can be converted to each other to meet the energy requirements of the metabolic network in an organism [44]. Glycolysis is an oxygen-independent metabolic pathway; *HK* promotes glucose phosphorylation to produce glucose-6-phosphate, the first step in the glycolytic pathway [45]. We observed that *HK* mRNA levels in group C and *PK* mRNA levels in group L were up-regulated, thereby confirming that *HK* activity can be regulated by the inhibitory effects of *PK*, which is the rate-limiting enzyme in glycolysis. *PK* catalyzes phosphoenolpyruvate conversion to adenosine diphosphate to produce pyruvate and adenosine triphosphate [46]; when ATP intracellular levels are high, *PK* inhibits *HK* activity, thereby limiting glucose phosphorylation [47]. These data showed that glycolytic processes were accelerated in *O. potamophila* larvae across the three groups and further accelerating energy supplies. Thus, we hypothesized that this may have been caused by an insufficient share of the energy supply from *D. magna* and *L. hoffmeisteri* as live prey.

*PEPCK* and *PK* are in a mutually complementary relationship in the gluconeogenic pathway; *PEPCK* maintains cellular energy homeostasis and glucose supply by catalyzing the first rate-limiting step of gluconeogenesis in the liver and by regulating phosphoenolpyruvate flow [48,49]. A possible mechanism could be that saturated and monounsaturated fatty acids in *H. molitrix* inhibited glycogen storage in *O. potamophila* larvae by decreasing protein kinase B phosphorylation. Also in gluconeogenesis, *GK* is the rate-limiting enzyme in the first reaction step that promotes glycogen synthesis and glucose glycolysis [50], and it was also highly expressed in group H. *LDH* is a lactate dehydrogenase involved in lactic-acid-producing reactions in the glycolytic pathway [51,52], with *LDH* mRNA expression levels and lactate production levels closely related to ATP demands and oxygen supply in cells.

In this study, we applied a TMT labeling proteomics approach to explore the molecular mechanisms underlying the effects of different live prey on *O. potamophila* larvae [23]. Our GO enrichment analyses on DEPs showed that they were mainly related to glycolytic processes, the endoplasmic reticulum lumen, NAD binding, intermediate filaments, and nutrient reservoir activity. Proteome sequencing data indicated that water meshworms regulated enzyme expression related to sugar metabolism in *O. potamophila* larvae, including phosphodiesterase, endoglucanase, and phosphoenolpyruvate carboxylase. These results suggested that *L. hoffmeisteri* mediated the digestion, absorption, and metabolism of glycosylated molecules and improved the regulation of energy metabolism in *O. potamophila* larvae.

The endoplasmic reticulum is one of the largest membrane systems in the cytoplasm and has a variety of functions, including protein synthesis and folding, lipid metabolism, and calcium ion storage [53]. The compositional and diverse network of molecules in *D. magna*, *L. hoffmeisteri*, and *H. molitrix* appeared to regulate endoplasmic reticulum composition in *O. potamophila* larvae. The inner lumen of the endoplasmic reticulum serves as a protein synthesis and protein-folding site and is an important focus of proteomics research [54]. Similarly, molecular chaperones in the endoplasmic reticulum lumen, including chaperones, calnexin, and calreticulin, help facilitate protein folding and assembly and regulate N-glycosylation modification reactions [55]. Our proteomics data analyses suggested that *H. molitrix* weakened protein assembly and modification in *O. potamophila* larvae relative to *L. hoffmeisteri*.

NAD is a widespread cofactor in living organisms and is synthesized in a multistep process from arginine, ornithine, and asparagine. The molecule is involved in several metabolic reactions, including sugar, fat, and amino acid metabolism [56], and regulates the glucose/lipid metabolism balance and homeostasis in *O. potamophila* larvae in conjunction with *FAS* and *HK*, among others. NAD structural domain proteins include dopamine β-oxidase and SIRT proteins, which facilitate post-translational modifications in proteins, cell metabolism and apoptosis, and gene regulation [57,58]. However, the effects of these molecules on immune and antioxidant properties in *O. potamophila* larvae remain to be investigated.

Intermediate filaments are a family of cytoskeletal proteins mainly including keratins, desmin, and glial fibrillary acidic protein [59], which are involved in biological processes, such as morphology maintenance, signaling and cell motility [60,61]. The effects of the mixed group on cytoskeletal/skeletal proteins in *O. potamophila* larvae were significantly higher when compared to group L, probably due to more comprehensive nutrition in terms of carbohydrates and fats. Studying nutrient reservoir activity can help identify important protein mechanisms in regulating nutrient metabolism in organisms. For example, the phosphorylation of sugar storage proteins, such as glycogen synthase and glycogenolytic enzymes [62,63], regulates glycogen synthesis and catabolic processes. During our glucometabolism analyses in *O. potamophila* larvae, it was evident that the mixed group caused more profound effects in larvae.

Other transcription factors, such as the peroxisome-proliferator-activated receptor family and the *SREBP* family, play important roles in energy and nutrient metabolism in organisms [64,65]. As mentioned, *SREBP-1* and *FAS* play key roles regulating fatty acid synthesis and homeostasis in *O. potamophila* larvae, and genes such as *ACOX1*, *ACOX3*, *HK*, and *PEPCK* appear to regulate the expression of lipid metabolism proteins and glucose synthase, which influence glycolipid metabolism networks in *O. potamophila* larvae liposomes. Intracellular signaling networks can also regulate the transport and release of nutrient reservoir proteins; for example, 5′ adenylate-activated protein kinase is affected by the energy status as it activates lipid and sugar storage proteins in low-energy states [66], thus promoting energy storage and metabolic balance [67,68].

Our KEGG enrichment analysis of DEPs revealed that arginine and proline metabolic pathways appear to interact with energy metabolism. In these metabolic pathways, intermediate metabolites can enter the tricarboxylic acid cycle or the lactic acid fermentation pathway to produce energy molecules, such as adenosine triphosphate or nicotinamide adenine dinucleotide [69], which can affect the energy supply and metabolic state in *O. potamophila* larvae.

## 5. Conclusions

Based on our molecular analyses and proteomics approach, we analyzed differentially expressed genes and DEPs related to in vivo glycolipid metabolism and energy metabolism in *O. potamophila* larvae after feeding them with different live prey and observed that different feeding combinations generated different response mechanisms. Our proteomics analyses also revealed that *O. potamophila* larvae fed a mix of all live prey (*D. magna*, *L. hoffmeisteri*, and *H. molitrix* fry) had better growth performance and more active pathway response mechanisms in terms of glycolipids and energy metabolism.

## Figures and Tables

**Table 1:** Primer sequences for target genes.

Primer	Sequence	Temperature
*FAS-F*	GGCAACAACACGGATGGATAC	55 °C
*FAS-R*	CTCGCTTTGATTGACAGAACAC	55 °C
*SREBP-1-F*	GAGCAAGTCTCTGAAGGATCTGGT	55 °C
*SREBP-1-R*	CCTCATCCACAAAGAAGCGGTG	55 °C
*ACOX1-F*	GATCATCGGCACCTACGCT	55 °C
*ACOX1-R*	TGACTGTGGGACTGTTCAAGAC	55 °C
*ACOX3-F*	CAGGGCAATTACTTGAGCG	55 °C
*ACOX3-R*	TTGAGGATGAAATCAGTGGGT	55 °C
*CPT1-α-F*	GCCTTTCAGTTCACCATCACA	55 °C
*CPT1-α-R*	ATGCGGCTGACTCGTTTCTT	55 °C
*HK-F*	GGGCATGAAAGGCGTGTC	55 °C
*HK-R*	TCTCCCTCGCAGCCTGAT	55 °C
*PK-F*	ACGGGTCGGTTATCTGGTTG	55 °C
*PK-R*	GCCTTTGCGACTTCCCAGA	55 °C
*LDH-F*	CGCCCTGGTGGATGTGAT	55 °C
*LDH-R*	CGATGCGGGAGTTTGCTG	55 °C
*PEPCK-F*	GGAGATGAGCTGGATGCAAATG	55 °C
*PEPCK-R*	CATCAAAGCTCTTGTGAACAA	55 °C
*GK-F*	ACAGAGTGGTGGACGAGACC	55 °C
*GK-R*	TCGTTCACCAGCTTCATCAG	55 °C
*β-Actin-F*	ATCGCCGCACTGGTTGTTGAC	55 °C
*β-Actin-R*	CCTGTTGGCTTTGGGGTTC	55 °C

**Table 2:** Growth performance and survival rate of *O. potamophila* larvae fed different live prey for 56 days.

Groups	IBW (g)	FBW (g)	WGR (%)	SGR (%)	SR (%)
C	0.14 ± 0.01	0.49 ± 0.03 ^a^	263.52 ± 31.48 ^a^	4.60 ± 0.31 ^a^	0.93 ± 0.01
L	0.14 ± 0.01	0.66 ± 0.02 ^b^	376.04 ± 31.23 ^b^	5.57 ± 0.23 ^b^	0.93 ± 0.01
H	0.14 ± 0.01	0.49 ± 0.02 ^a^	262.27 ± 35.60 ^a^	4.58 ± 0.36 ^a^	0.92 ± 0.01
M	0.14 ± 0.01	0.71 ± 0.04 ^c^	410.10 ± 25.05 ^b^	5.82 ± 0.18 ^b^	0.93 ± 0.01

The same letters over columns indicate non-significant differences (*p* > 0.05). Significant differences from the control group (group M) are indicated by different letters (*p* < 0.05).

## Data Availability

The data presented in this study are contained within the article.
